# Impact of Nox5 Polymorphisms on Basal and Stimulus-Dependent ROS Generation

**DOI:** 10.1371/journal.pone.0100102

**Published:** 2014-07-03

**Authors:** Yusi Wang, Feng Chen, Brian Le, David W. Stepp, David J. R. Fulton

**Affiliations:** 1 Vascular Biology Center, Medical College of Georgia at Georgia Regents University, Augusta, Georgia, United States of America; 2 Department of Forensic Medicine, Nanjing Medical University, Nanjing, Jiangsu, China; 3 Department of Physiology, Georgia Regents University, Augusta, Georgia, United States of America; 4 Department of Pharmacology, Georgia Regents University, Augusta, Georgia, United States of America; University of California, Merced, United States of America

## Abstract

Nox5 is an EF-hand containing, calcium-dependent isoform of the NADPH oxidase family of reactive oxygen species (ROS) generating enzymes. Altered expression and activity of Nox5 has been reported in cardiovascular diseases and cancers but the absence of Nox5 in rodents has precluded a greater understanding of its physiological and pathophysiological roles. Multiple polymorphisms have been identified within the coding sequence of human Nox5, but whether this translates into altered enzyme function is unknown. Herein, we have generated 15 novel mutants of Nox5β to evaluate the effect of exonic SNPs on basal and stimulated enzyme activity. Compared to the WT enzyme, ROS production was unchanged or slightly modified in the majority of mutants, but significantly decreased in 7. Focusing on M77K, Nox5 activity was dramatically reduced in unstimulated cells and following challenge with both calcium- and phosphorylation-dependent stimuli despite equivalent levels of expression. The M77K mutation did not influence the Nox5 phosphorylation or the ability to bind Hsp90, but in cell-free assays with excess co-factors and calcium, ROS production was dramatically reduced. A more conservative substitution M77V arising from another SNP yielded a different profile of enzyme activity and suggests a critical role of M77 in calcium-dependent ROS production. Two C-terminal mutants, R530H and G542R, were observed that had little to no activity and relatively high minor allele frequency (MAF). In conclusion, we have identified 7 missense SNPs in Nox5 that result in little or no enzyme activity. Whether humans with dysfunctional Nox5 variants have altered physiology or disease remains to be determined.

## Introduction

The NADPH oxidases (Nox1–5) comprise a family of structurally related membrane-spanning oxidases. Nox enzymes are major sources of reactive oxygen species (ROS), including superoxide and hydrogen peroxide, in multiple cell types [1]. ROS have important and well established roles in physiology and in the pathogenesis of many diseases including cancer, hypertension and atherosclerosis. Nox2 is primarily expressed in immune cells such as neutrophils and macrophages and loss of function mutations in Nox2 or its regulatory subunits leads to impaired immune cell function that manifests as chronic granulomatous disease [2], [3]. Nox3 expression is restricted to the inner ear and has an important developmental role and genetic deletion results in the impaired formation of otoconia and a head tilt phenomenon [4], [5]. The other Nox enzymes (Nox1, 4–5) are primarily expressed in cells outside of the immune system and have functions that are less well defined. Nox1 is expressed in colon epithelium and smooth muscle and genetic deletion of Nox1 has no overt baseline phenotype but results in altered disease susceptibility including some forms of hypertension [6]–[8], vascular remodeling [9] and atherosclerosis [10]. Coding polymorphisms Nox1 (H315R) are associated with diabetic nephropathy [11]. Nox4 is more ubiquitously distributed with higher levels of expression in the kidney and blood vessels. Nox4 knockout mice are phenotypically normal with altered disease susceptibility [12]. However, compared to the other Nox isoforms, little is known about the functional significance of Nox5. A major obstacle has been that the genomes of rats and mice do not encode Nox5 suggesting that Nox5 confers no selective advantage or that functions performed by Nox5 overlap with that of other Nox isoforms. In humans, Nox5 is expressed in the lymph nodes and testes with lower levels detected in blood vessels and other cells [13]. Higher levels of Nox5 expression have been observed in cancers [14]–[16] and cardiovascular disease [17] suggesting it may have important roles in the pathogenesis of human disease. Indeed, Nox5 expression is elevated in hypertension [18], post myocardial infarction [19], atherosclerosis [17] and diabetes [11], [20] and may play an important role in human cardiovascular disease. The expression of Nox5 is also increased in cancers and may contribute to enhanced cellular proliferation and resistance to apoptosis [14], [15], [21].

ROS production from Nox1–3 is controlled by protein: protein interactions and Nox4 is thought to be constitutively active [22]. Nox5 is unique for its absolute dependence on calcium which binds to four N-terminal EF hands. Nox5 activity is thus intimately linked to the local calcium concentration [23] and extracellular agonists that mobilize intracellular calcium robustly stimulate Nox5 activity. In addition, Nox5 activity can also be modulated by increasing its calcium-sensitivity through PKC- and other kinase-dependent phosphorylation [24]. PMA-dependent phosphorylation of Nox5 occurs on Ser^490^ and Thr^494^ and Ser^498^ and loss of phosphorylation impairs calcium-dependent ROS production. Nox5 activity and expression can also be regulated by binding to proteins such as Hsp70, Hsp90 and calmodulin [25]. Increased expression of Nox5 can impair endothelium-dependent relaxation [26] and alter cellular proliferation [27]. Whether nonsynonymous SNP influence the ability of Nox5 to produce ROS is not yet known.

Nox5 was the last of the conventional Nox enzymes to be identified [13] and genetically is the most divergent isoform [28]. The cDNA encodes a unique N-terminal calcium binding domain that regulates calcium-dependent activity, a central 6 transmembrane oxidoreductase that has significant structural homology to the other Nox isoforms and a C-terminal flavin and NADPH binding domain [13]. In humans, the gene for Nox5 is located on chromosome 15 and at least five known splice variants of Nox5- α, β, γ, δ and a truncated variant (Nox5-Short, -S or ε) have been described [13]. These have also been denoted NOX5v1-v5 [29] The primary differences between these variants are modifications of the N-terminus which is vital for calcium binding and catalytic activity. Recent studies suggest that only 2 of these isoforms are functional, but there have been conflicting reports in cells with native expression of the short isoform [30]. The Nox5 gene is highly polymorphic with numerous intronic and exonic single nucleotide changes [29]. Indeed within the coding region of Nox5 there are approximately 100 reported nonsense, missense and synonymous SNPs (NCBI) with frequencies varying from MAF (Minor Allele Frequency) 0.0005 to 0.31. Some of the missense SNPs occur in regions of Nox5 that may impact function including EF hands, transmembrane regions and the NADPH binding C-terminus. One validated SNP (rs34406284) and two non-validated SNPs (rs369517329, rs370082662) encode nonsense mutations resulting in truncated Nox5 enzymes that cannot function, essentially Nox5 knockouts.

The primary goal of the current study is to investigate the relationship between SNPs in the coding region of Nox5 and possible changes in enzyme activity. To this end, we have generated mutants of Nox5 that are either the most frequent or those which occur in regions most likely to impact activity. The ability of these mutants to generate ROS was compared to the WT enzyme under basal conditions and following both calcium and phosphorylation dependent activation. Results from these experiments may have important implications as loss or gain of function changes in Nox5 may be linked to altered susceptibility to disease and thus provide valuable insights into the importance of Nox5 to human health.

## Methods

### Cell Culture and Transfection

COS-7 cells [31], [32] were purchased from ATCC and cultured in Dulbecco’s modified Eagle’s medium (Invitrogen) containing L-glutamine, penicillin, streptomycin, and 10% (v/v) fetal bovine serum. Cells were transfected using Lipofectamine 2000 according to the manufacturer’s instructions (Invitrogen).

### DNA Constructs

The cDNA encoding Nox5β contains an N-terminal HA tag. Mutations corresponding to SNPs were introduced by PCR using WT-Nox5β cDNA as the template and the primers listed in **Table 1**. DNA sequences were confirmed by automated sequencing and expression verified by Western blotting.

**Table 1:** Sequences of Nox5 mutagenic primers.

	Mutations	Primers
1	M77V	5′-CAGCCCCGTGGACAAACTCAAATTCCTCTTCC-3′
2	M77K	5′-GCCCCAAGGACAAACTCAAATTCCTCTTCCAG-3′
3	K79I	5′-CCATGGACATCCTCAAATTCCTCTTCCAGGTGTATG-3′
4	P97A	5′-ATTGACGCCGATGAGCTGCGCACTGTGCTGCAGTC-3′
5	S236R	5′-CGACTGCCGCTTCATCGCGGTGCTGATGCTCAG-3′
6	T253M	5′-GCGGGCCACGTGGCTGGCTCAAGTCCTACCACTGG-3′
7	L334F	5′-CTGCTCCTCTTCATGTTCATCTGCTCCAGTTCCTG-3′
8	L360P	5′-CCTACCCCCTCGTGTGGCTTCTGCTCATCTTTC-3′
9	L361P	5′-CTACCTCTTCGTGTGGCTTCTGCTCATCTTTCATG-3′
10	R419Q	5′-ATCAAGCAGCCCCCTTTTTTTCACTATAGACCTG-3′
11	R530H	5′-GACCCCCACCCACAGGATCTTTGCCTCTGAGCATGCC-3′
12	G542R	5′-GCTCATCAGGGCAGGCATCGGCATCACCCCC-3′
13	K688E	5′-GAAGGGCGAGGTGCAGGTCTTCTTCTGTGGCTC-3′
14	V689A	5′-GCAAGGCCCAGGTCTTCTTCTGTGGCTCCCCAG-3′
15	R713G	5′-CGGCTTCGGATTTTTCCAAGAGAATTTCTAGC-3′

### Measurement of ROS

COS-7 cells were transfected with cDNAs encoding WT HA-Nox5β or the various mutants of HA-Nox5β. 24 h later, cells were detached and either replated into 96-well plates (Thermo Lab systems) at a density of ∼5×10^4^ cells/well or processed for expression analysis via Western Blot. Cell media was changed to phenol-free Dulbecco’s modified Eagle’s medium (Sigma) containing 400 µM concentration of the luminol analogue L-012 (Wako) and incubated for 30 min prior to the addition of agonists. Luminescence was quantified over time using a Lumistar Galaxy (BMG) luminometer. The specificity of L-012 for ROS was confirmed by transfecting cells with a control plasmid such as green fluorescent protein, lacZ, or by co-incubation of a superoxide scavenger, such as Tiron (5 mmol/L) [24]. Hydrogen peroxide was measured using the Amplex Red assay with excitation at 544 nm and emission detection at 590 nm. Cells were incubated for 20 min at 37°C with 50 µM Amplex Red and 0.125 U/ml HRP. In all cases, background levels of hydrogen peroxide obtained from mock-transfected controls (lacZ) were subtracted from that obtained from cells expressing Nox enzymes [33].

### Co-Immunoprecipitation and Western Blotting

Cells were lysed on ice in a lysis buffer containing 20 mM Tris-HCl (pH 7.4), 137 mM NaCl, 1% NP-40, 10 mM NaF, 10% Glycerol, 0.1 mM depolymerized sodium vanadate, and 1% protease inhibitor cocktail (Sigma). SDS-PAGE and immunoblotting were performed using standard procedures. In brief, 500 µg of cell extract was incubated for 2 h at 4°C with the relevant antibody: anti-HA (Roche Applied Science), anti-Hsp90(BD) and complexes were precipitated with A/G beads (Santa Cruz). Antigens were detached from the beads by incubating with SDS sample buffer at 95°C for 5 min and proteins were subjected to Western blot analysis. Nox5 phosphorylation state specific antibodies to Ser490, Thr494, and Ser498 were generated by Pacific Immunology as previously described [33].

### Cell-free Activity Assays

COS-7 cells expressing WT and mutant Nox5 were lysed in a MOPS (30 mM, pH 7.2)-based buffer containing KCl (100 mM), Triton (0.3%), and protease inhibitors (Sigma). Adherent cells were rocked gently, and the lysis buffer was aspirated and then washed three times with phosphate-buffered saline (4°C). Remaining cytoskeletal fractions were resuspended in MOPS buffer containing 3 mM EGTA to remove any residual calcium. These fractions were sonicated at low power and pelleted by centrifugation at 14,000 rpm (4°C). The supernatant was then aspirated, and the pellet was resuspended in MOPS buffer with mild sonication. The cell-free extract was aliquoted into buffers containing L012 (400 µM), 1 mM MgCl2, 100 µM FAD (Sigma), and buffered free calcium at 0 and 26 µM (Invitrogen/Molecular Probes calcium calibration buffer kit). After a brief period of equilibration, reduced NADPH (Sigma) was injected to a final concentration of 200 µM, and the production of reactive oxygen species was monitored over time [24].

### Statistical Analysis

Luminescence data are expressed as mean ± S.E. Statistical analysis was performed using GraphPad InStat software (GraphPad Software, Inc.) and comparisons between two groups (paired WT and mutant) were made using a two-tailed Student’s *t*-test. Differences were considered as significant at *p<*0.05 (*).

## Results

### Effect of Nox5 Mutations on Basal and Stimulated ROS Production

**Figure 1:**
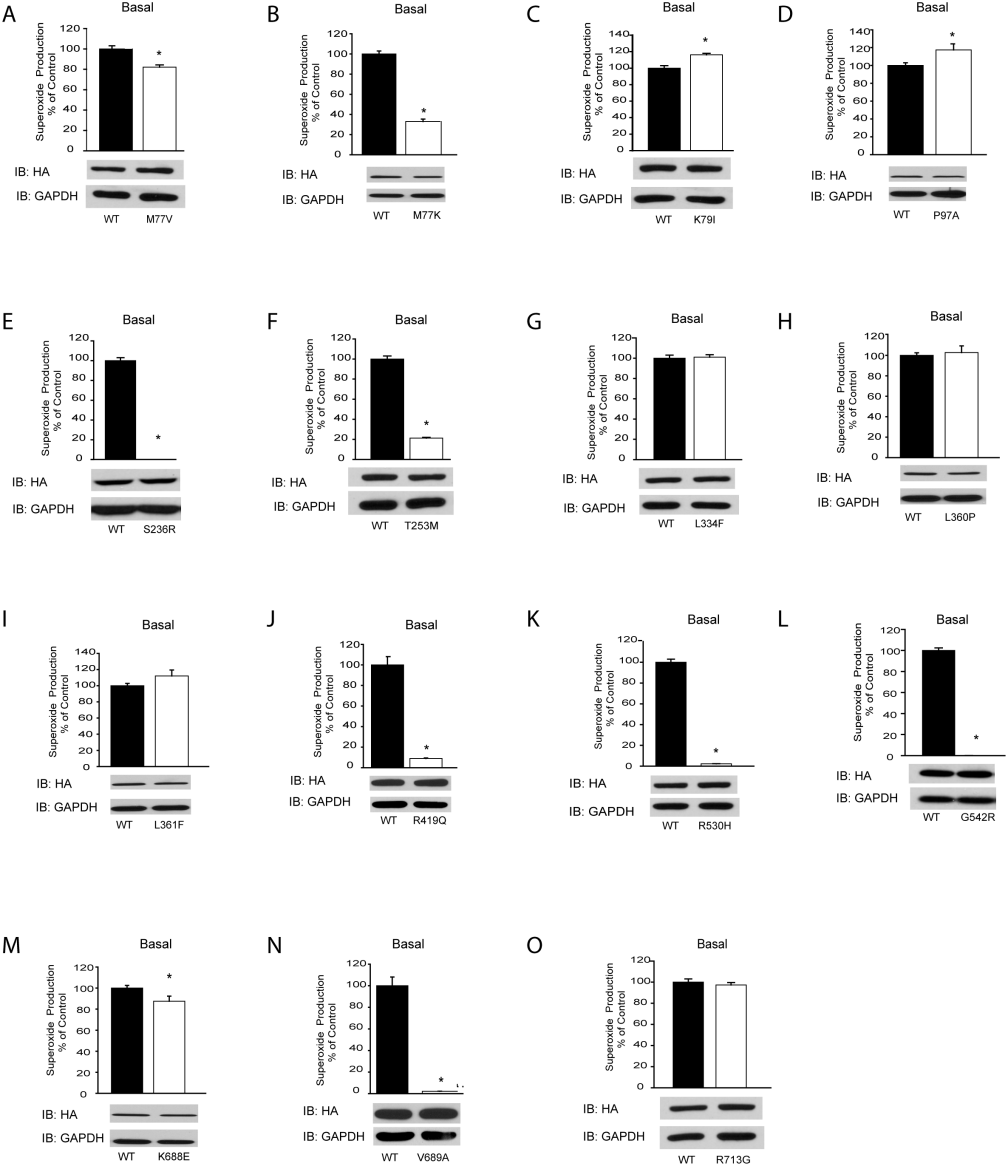
Basal superoxide production from Nox5 mutants. Superoxide production was monitored using L-012 in COS-7 cells transfected with HA-Nox5 or mutants based on SNPs listed in **Table 2** (A–O). Upper panel shows unstimulated or basal superoxide release from HA-Nox5 or the mutants. Western blots in the lower panels reveal full length protein expression of transgenes versus the loading control, GAPDH. Results are presented as mean ± SEM (n = 4–6), *P<0.05, *versus* HA-Nox5.

To assess the effect of exonic SNPs (**Table 2**) on Nox5 activity and ROS production, we used chemiluminescence to measure superoxide production in untreated cells (basal) or following stimulation with a calcium-dependent agonist (ionomycin) or an agonist that increases activity via changes in Nox5 phosphorylation (PMA). As shown in **Figs. 1** **and** **2**, the majority of SNPs were well tolerated by Nox5 with no major changes in basal activity. However, 7 out of 15 had a significant loss of activity despite equal levels of enzyme expression. S236R, G542R, V689A mutants were completely inactive both under unstimulated conditions and following ionomycin and PMA challenge (**Figs. 1** **,** **2**). T253M, R419Q, R530H had measurable basal and stimulated activity that was <10% of WT Nox5. We did not observe any mutants with robust increases in activity although some, including K79I and P97A, had statistically significant increases in activity that were regarded as minor. Western Blot was performed on all transfected cells to confirm that the same amount of Nox5 protein was expressed (**Fig. 1**, lower panels). Whether the bulk subcellular localization of Nox5 was altered by the M77K substitution was assessed by confocal imaging of GFP-Nox5 in live cells. We found that GFP-Nox5 and GFP-Nox5 M77K had a similar subcellular distribution (**Figure S1**) suggesting that this mutation does not have a major influence on the intracellular location of Nox5.

**Figure 2:**
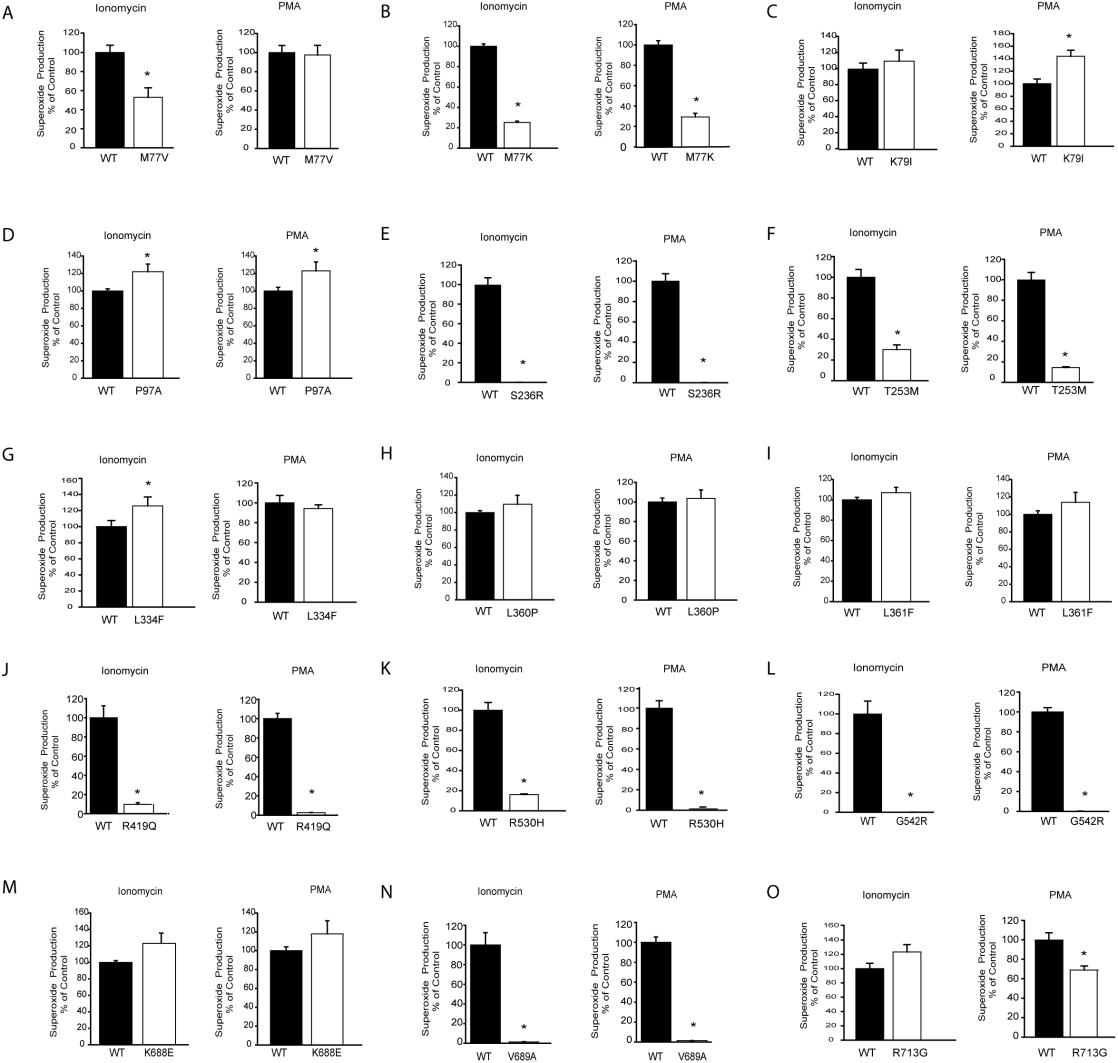
Stimulated superoxide production from Nox5 mutants. Stimulated superoxide production was monitored in COS-7 cells transfected with HA-Nox5 or mutants via L-012 chemiluminescence (A–O). Superoxide release was monitored over time from cells stimulated with ionomycin (1 µM) or PMA (100 nM). Maximal superoxide produced is presented as mean ± SEM (n = 4–6), *P<0.05, *versus* HA-Nox5.

**Table 2:** Nonsense and missense single nucleotide polymorphisms within the gene coding region of Nox5. Position numbers refer to AF325189.1

Chr. Position	mRNA pos.	dbSNP rs#	Heterozygosity	MAF	Function	RefSNP Alleles	Protein residue	Amino acid pos.	MAF (%) (EA/AA/All)*	Conserved*
69318938	368	rs369517329	N.D.		**nonsense**	G/T (FWD)	Glu[E]/Ter[*]	23	0.0116/0.0/0.0077	Yes
69320663	530	rs34097994	0.005	0.0023	missense	A/G (FWD)	Met[M]/Val[V]	77	0.0/1.0682/0.3616	Yes
69320664	531	rs112069106	0.001	0.0005	missense	A/T (FWD)	Met[M]/Lys[K]	77	0.0/0.1364/0.0462	Yes
69320670	537	rs36036826	0.004	0.0018	missense	A/T (FWD)	Lys[K]/Ile[I]	79	0.0/0.9091/0.3078	Yes
69323959	590	rs78571685	0.046	0.0234	missense	C/G (FWD)	Pro[P]/Ala[A]	97	0.0233/0.1364/0.0616	No
69325606	1007	rs150003957	0.030	0.0152	missense	A/C (FWD)	Ser[S]/Arg[R]	236	0.9078/1.6598/1.1624	Yes
69327734	1059	rs145609289	0.007	0.0037	missense	C/T (FWD)	Thr[T]/Met[M]	253	0.0/1.2045/0.4078	Yes
69327737	1062	rs34406284	0.0360	0.0184	**nonsense**	A/G (FWD)	Trp[W]/Ter[*]	254	0.0/8.3182/2.8163	Yes
69328226	1301	rs12907196	0.427	0.3090	missense	C/T (FWD)	Leu[L]/Phe[F]	334	44.4858/17.1818/42.5362	Yes
69329396	1380	rs78158861	N.D.		missense	C/T (FWD)	Leu[L]/Pro[P]	360		Yes
69329398	1382	rs77073843	N.D.		missense	C/T (FWD)	Leu[L]/Phe[F]	361		No
69331219	1557	rs117880022	0.001	0.0005	missense	A/G (FWD)	Arg[R]/Gln[Q]	419		Yes
69331323	1661	rs370082662	N.D.		**nonsense**	C/T (FWD)	Gln[Q]/Ter[*]	454	0.0/0.0227/0.0077	Yes
69339787	1890	rs2277552	0.189	0.1056	missense	C/T (REV)	Arg[R]/His[H]	530	0.2908/11.2727/4.0089	Yes
69339822	1925	rs188055665	0.030	0.0152	missense	A/G (FWD)	Gly[G]/Arg[R]	542	0.0349/0.0/0.0231	Yes
69348938	2363	rs112389647	0.500		missense	A/G (FWD)	Lys[K]/Glu[E]	688		Yes
69348942	2367	rs113164499	0.003		missense	C/T (FWD)	Val[V]/Ala[A]	689	0.0/0.2955/0.1	Yes
69349013	2438	rs7168025	0.017	0.0087	missense	A/G (FWD)	Arg[R]/Gly[G]	713	0.0/3.25/1.1003	Yes

*EA: European Ameriacan.

*AA: African American.

*Reference species: *Homo sapiens, Bos taurus, Canis lupus familiaris, Oryctolagus cuniculus, Equus caballus, Macaca mulatta, Gorilla gorilla, Ovis aries, Felis catus and Sus scrofa.*

### Investigation of Mechanisms Underlying the Reduced Activity of the M77K Nox5 Mutant

**Figure 3:**
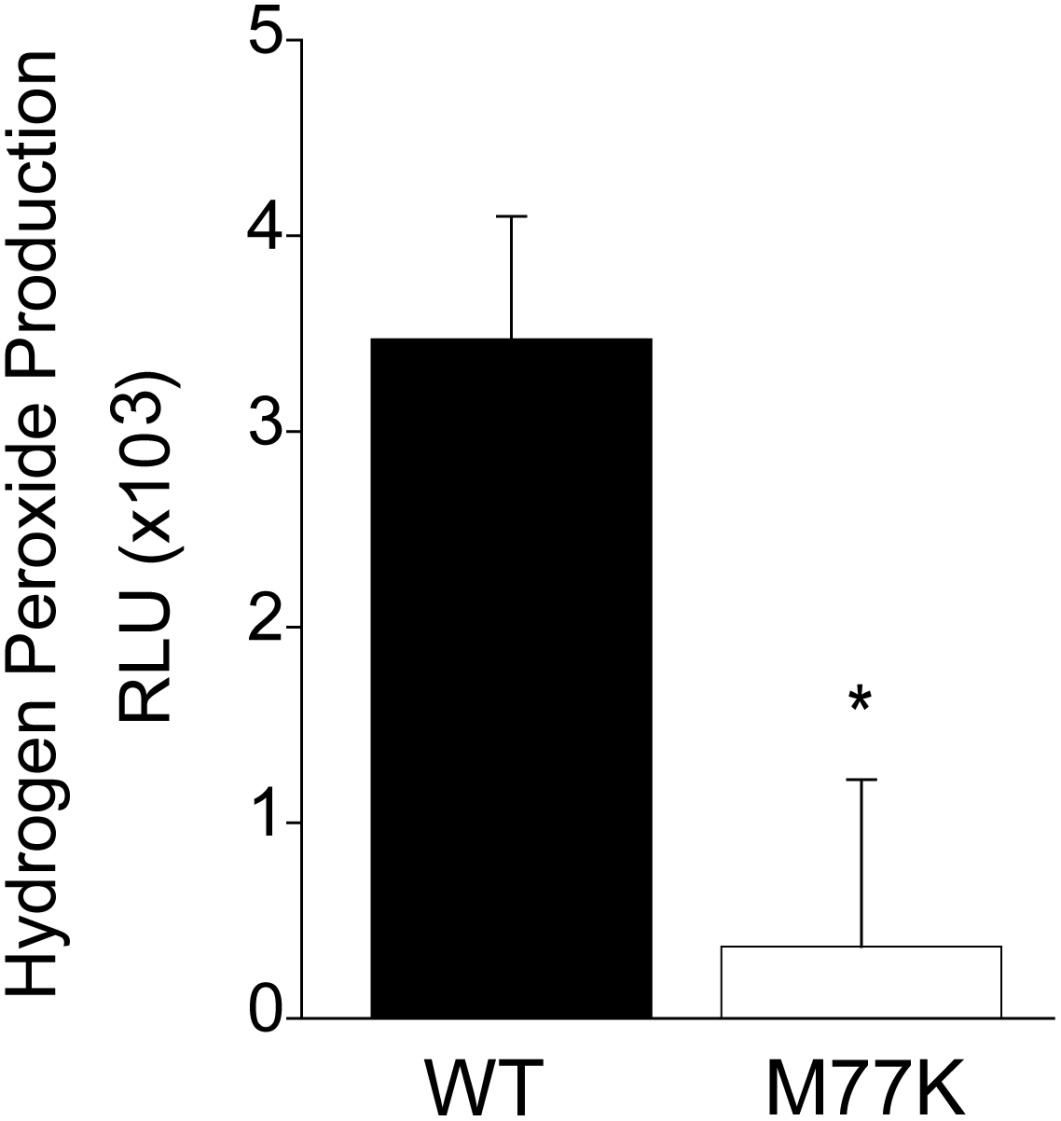
Hydrogen peroxide production from Nox5 versus the M77K mutant. COS-7 cells were transfected with HA-Nox5 or the mutant M77K. Cells were incubated for 20 min at 37°C with 50 µM Amplex Red and 0.125 U/ml HRP. Hydrogen peroxide was measured via background subtracted Amplex red fluorescence in cells transfected with a control plasmid (LacZ). Results are presented as mean ± SEM (n = 4–6), *P<0.05, *versus* HA-Nox5.

**Figure 4:**
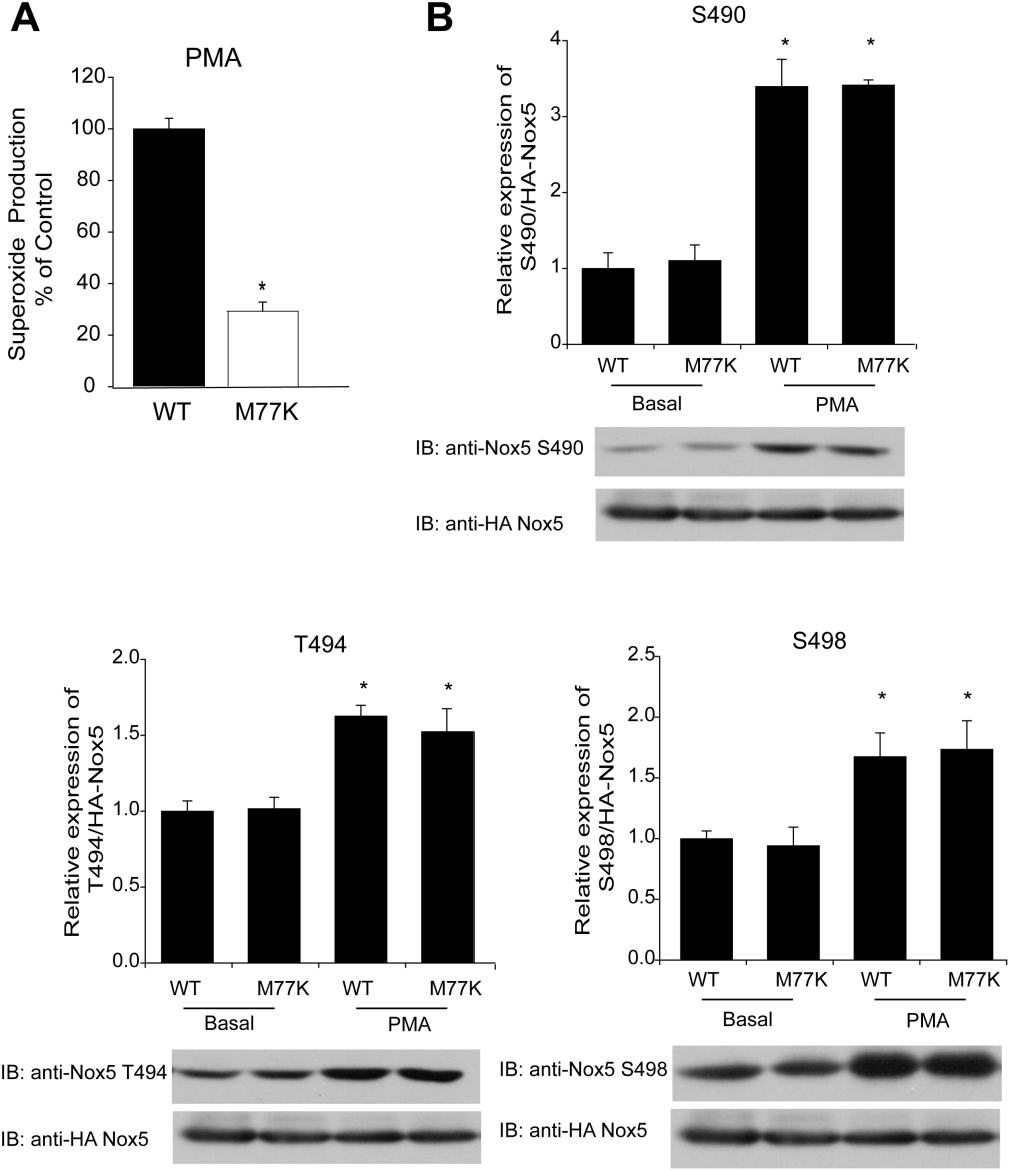
The M77K mutant does not influence Nox5 phosphorylation. (**A**) Stimulated superoxide production was measured over time from COS-7 cells expressing HA-Nox5 or the mutant M77K following the addition of PMA (100 nM). Results show the maximum level of superoxide produced and are presented as mean ± SEM (n = 4–6), *P<0.05, versus HA-Nox5. (**B**) COS-7 cells expressing HA-Nox5 or the mutant M77K with vehicle or PMA (100 nM) after 2 h serum starvation and the phosphorylation of Nox5 at Ser490, Thr494 and Ser498 were determined by Western blot using phosphorylation state specific antibodies relative to total Nox5. Quantitation and representative blots from 3 independent experiments are shown. *P<0.05, *versus* Basal WT-Nox5.

The M77K Nox5 mutant had reduced capacity to produce superoxide under basal (**Fig. 1**) or stimulated (**Fig. 2**) conditions. Exploring further, we investigated whether there were changes in the profile (superoxide versus hydrogen peroxide) of ROS produced. As shown in **Fig. 3**, hydrogen peroxide production from Nox5 M77K was significantly reduced which is in line with the decreases observed in superoxide production. The ability of Nox5 M77K to produce ROS in response to PMA stimulation was also compared with WT Nox5 (**Fig. 4A**). The phosphorylation of Nox5 at Ser490, Thr494, and Ser498 in response to PMA has been shown to influence calcium-dependent activity by increasing sensitivity to lower levels of calcium. This mechanism has been shown to be important for basal, PMA and low level calcium-dependent production of superoxide [24]. Using phosphorylation-state specific antibodies we found that basal and stimulated phosphorylation of Nox5 was not different in the M77K mutant versus the WT (**Fig. 4B**). These results suggest that the M77K mutation does not influence basal or PMA-stimulated Nox5 phosphorylation.

**Figure 5:**
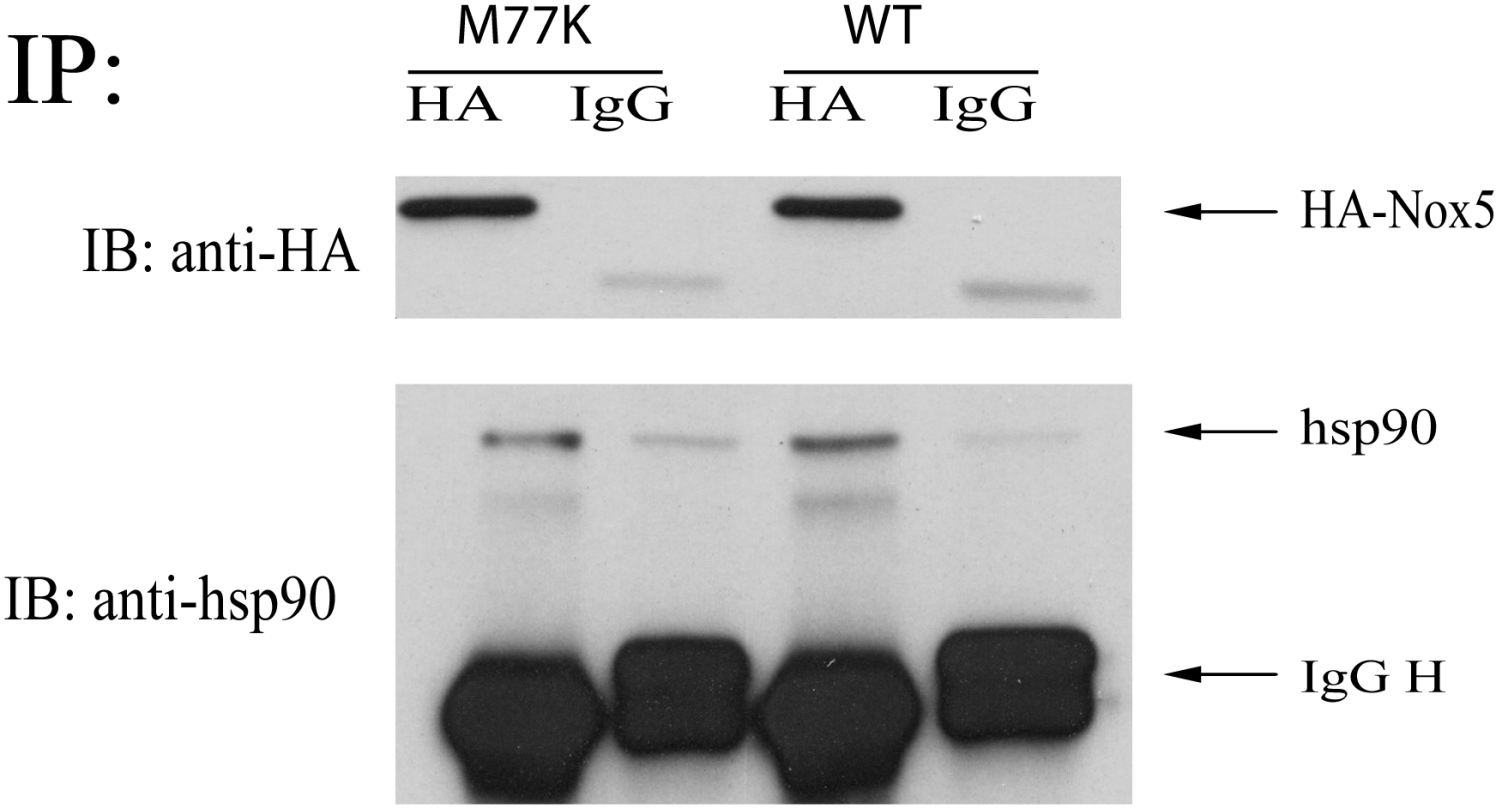
The M77K mutant does not influence hsp90 binding ability to Nox5. COS-7 cells were transfected with HA-Nox5 and M77K Nox5. 48 hrs later, Nox5 was immunoprecipitated from cell lysates using a monoclonal antibody against HA or a negative isotype control mouse immunoglobulin (IgG). Immune complexes were immunoblotted for the amount of Nox5 recovered (HA) and associated hsp90. Representative blots from 2–3 independent experiments are shown.

Previous studies by our lab have shown that hsp90 binding to Nox5 influences enzyme stability and superoxide production [25], [33] To investigate whether the M77K mutation influences the ability to bind hsp90, we performed co-immunoprecipitation using HA to immuno-isolate Nox5 and a hsp90 antibody to determine the amount of bound hsp90. At the same level of expression of M77K and WT Nox5, we observed no changes in hsp90 binding to Nox5 (**Fig. 5**).

### The M77K Mutation Hinders the Calcium-Dependent Activity of Nox5

**Figure 6:**
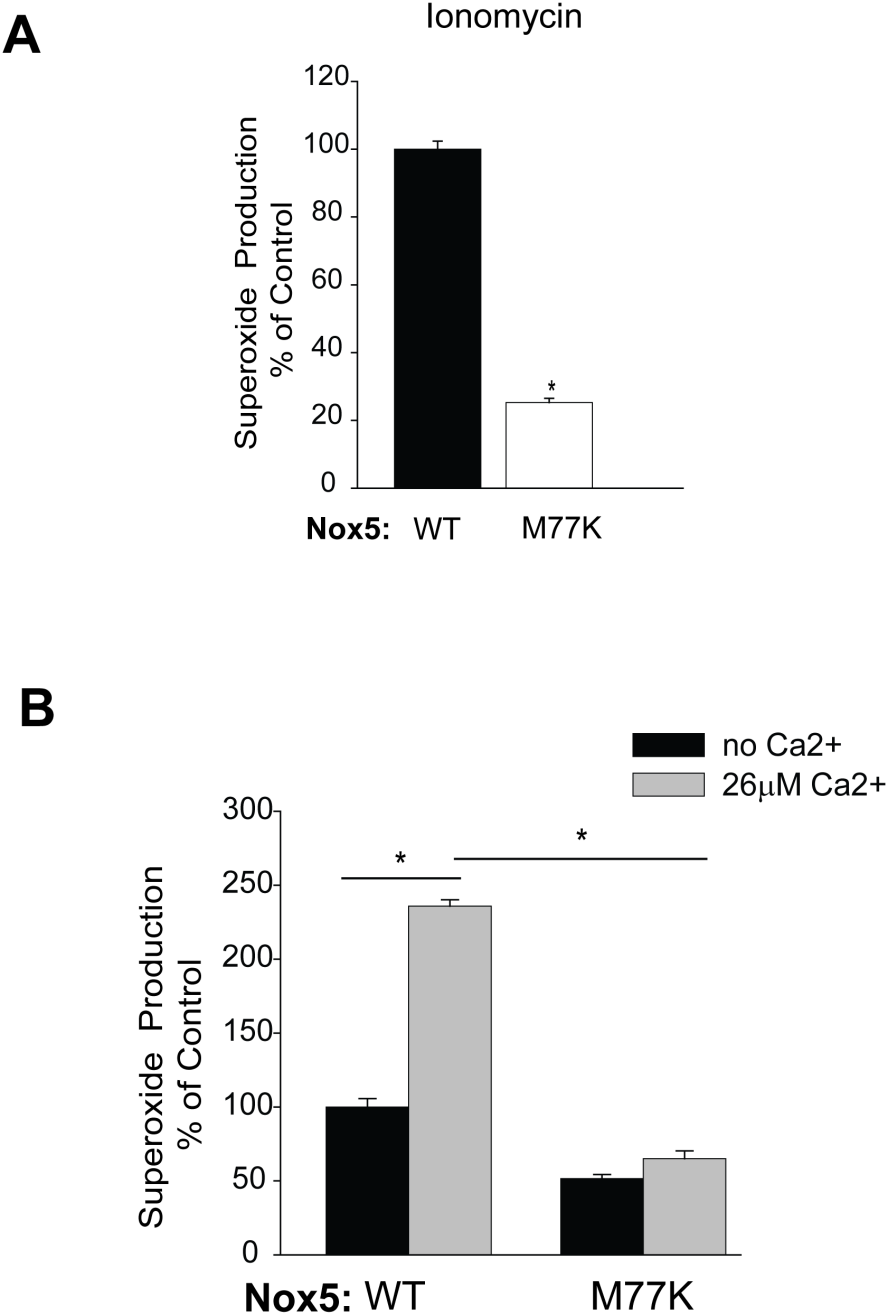
The M77K mutation decreases the calcium-induced activation of Nox5. (**A**) Stimulated superoxide release was measured over time from COS-7 cells expressing HA-Nox5 or the mutant M77K following addition with ionomycin (1 µM). Results show the maximum level of superoxide produced and are presented as mean ± SEM (n = 4–6), *P<0.05, versus HA-Nox5. (**B**) The activity of Nox5 in cell free extracts was determined in the absence or in the presence of free calcium (mean ± SEM n = 4). *P<0.05, *versus* HA-Nox5.

In response to the calcium-dependent agonist, ionomycin, superoxide production from M77K was significantly reduced compared to WT Nox5 (**Fig. 6A**). To explore if the M77K mutation constrains the ability of Nox5 to respond to calcium, we next performed a cell-free activity assay. Superoxide production was measured in the presence of excess co-factors, with and without a maximal concentration of buffered free Ca^2+^ [24]. Superoxide production from WT Nox5 was robustly increased in the presence of 26 µM Ca^2+^. However, the mutant M77K Nox5 was insensitive to high levels of Ca^2+^ as evidenced by the dramatically reduced production of superoxide at equal levels of expression (**Fig. 6B**). To further explore the mechanisms underlying the reduced activity of M77K, we generated another mutation, M77V representing a distinct SNP with a more conservative amino acid substitution. In contrast to the M77K Nox5, M77V had only slightly reduced basal activity and no change in PMA activity (**Fig. 1** **,** **Fig. 2A–B**). However, ionomycin-stimulated calcium-dependent activity was only ∼50% of the WT (**Fig. 2A–B**). These results suggest that M77 influences the ability of Nox5 to respond to calcium.

**Figure 7:**
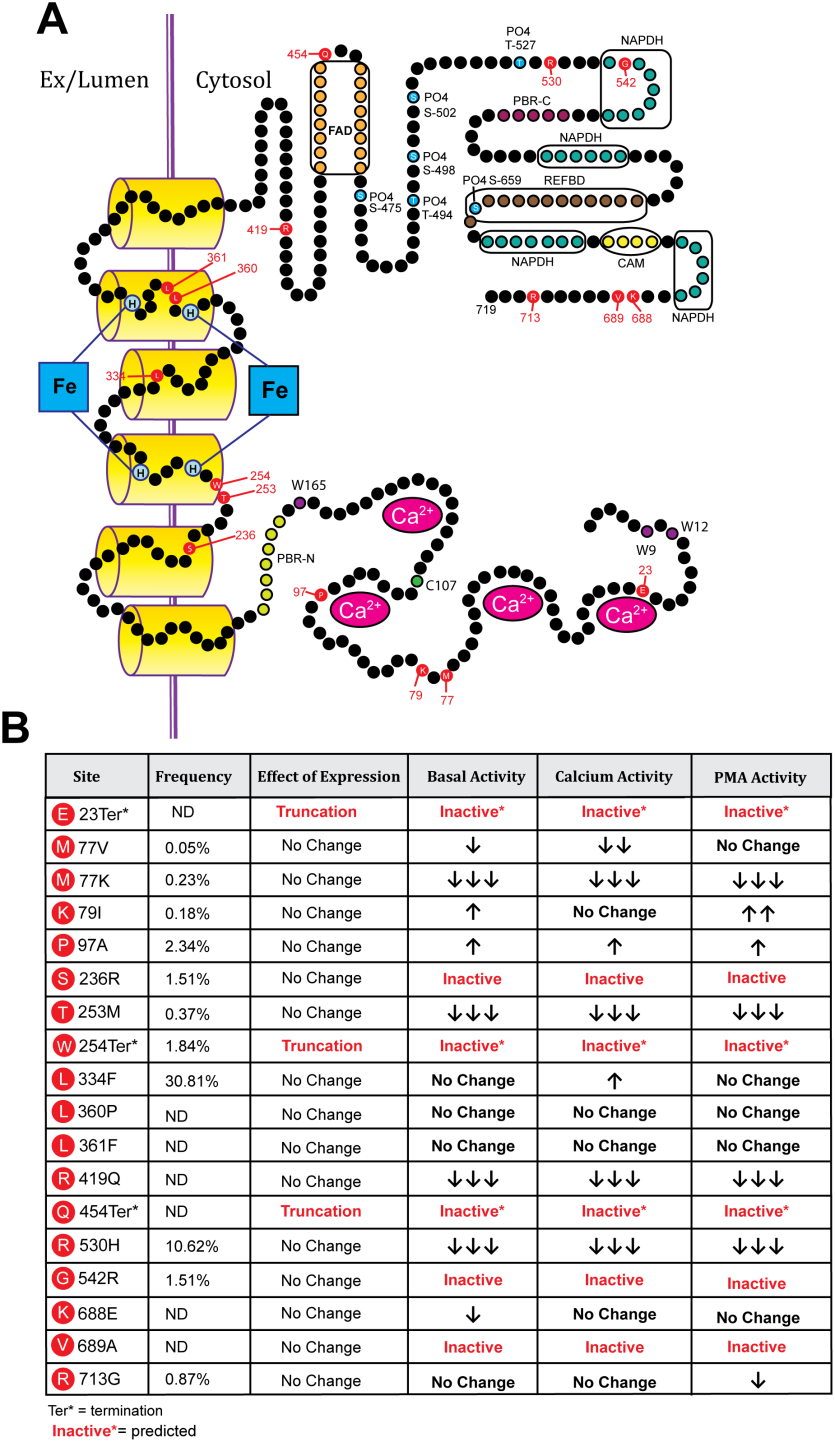
Overview of (A) the relative positions of the non-synonymous SNPs encoding amino acid mutations within Nox5 and (B) the relative frequency of these SNPs and the effect on basal, and ionomycin and PMA-stimulated activity.

The relative positions and functional effects of polymorphisms on Nox5 are summarized in **Fig. 7**.

### The Relevance of Other Single Nucleotide Polymorphisms of Nox5

The coding region of Nox5α has ∼108 reported coding SNPs of varying frequencies (http://www.ncbi.nlm.nih.gov/SNP/snp_ref.cgi?locusId=79400). Our study was primarily focused on identifying the SNPs that modify the amino acid sequence of Nox5 and potentially influence its activity. However, a recent report has indicated that certain codons may serve a dual purpose (duons) by also regulating the level of gene expression by enabling transcription factor binding within exons. Duons are highly conserved and it has been reported that SNPs within duons can significantly alter TF binding [34]. Relevant to our study, SNPs that are silent and do not change the amino acid sequence may indeed have relevance by influencing Nox5 gene expression through the creation or elimination of duons. Therefore we have listed the synonymous SNPs within Nox5 and determined whether the modified residue is conserved in the gene for Nox5 in other mammals (**Table 3**). Given the high degree of conservation observed, it is likely that duons exist within the Nox5 gene, and are possibly altered by SNPs to influence gene expression levels [35]. At present there is limited information relating genetic polymorphisms of Nox5 to disease susceptibility in different populations. With the knowledge gained from the changes in activity of Nox5 mutants arising from SNP, we compared the frequencies of these mutations based on the 1000 Genome Projects database. The 5 listed SNPs are those introducing mutations in Nox5 that alter activity significantly including M77K, S236R, T253M, W254Ter* and R530H and those for which data is available. An interesting finding is that the SNP encoding R530H has a relatively high frequency among Asians and Africans versus Europeans with South Americans being intermediate (0.115 African Americans, 0.208 Kenya, 0.119 Nigerians and 0 for Western and Northern Europeans, 0 from Great Brittain, Italy and Spain and 0.086 for Mexicans, 0.046 for Puerto Ricans, 0.067 Columbians). The W254Ter was predominantly found in Africans (0.074 in African Americans, 0.0625 in Kenyans and 0.108 in Nigerians) versus 0 in other populations (**Table 4**).

**Table 3:** Synonymous single nucleotide polymorphisms in the gene coding region of Nox5. Position numbers refer to AF325189

Chromosome Pos.	mRNA pos.	dbSNP rs#	Heterozygosity	MAF	Function	RefSNP Alleles	Protein residue	Aminoacid pos	MAF (%) (EA/AA/All)*	Conserved*
69320617	484	rs200419456	0.0030		synonymous	C/T (FWD)	Leu[L]/Leu[L]	61	0.0349/0.0/0.0231	Yes
69325386	787	rs201398847	0.0020		synonymous	C/T (FWD)	Ala[A]/Ala[A]	162		Yes
69325581	982	rs311893	0.2900	0.1763	synonymous	A/G (REV)	Cys[C]/Cys[C]	227	4.188/32.772/13.8568	Yes
69327753	1078	rs141118221	0.0000		synonymous	A/G (FWD)	Leu[L]/Leu[L]	259	0.0116/0.0/0.0077	Yes
69327825	1150	rs311889	0.2890	0.1754	synonymous	A/G (REV)	Thr[T]/Thr[T]	283	4.2578/33.1818/14.0505	Yes
69327828	1153	rs374520151	N.D.		synonymous	A/G (FWD)	Val[V]/Val[V]	284	0.0116/0.0/0.0077	Yes
69328180	1255	rs143898169	0.0030	0.0014	synonymous	C/T (FWD)	His[H]/His[H]	318		Yes
69329397	1381	rs370319410	N.D.		synonymous	C/T (FWD)	Leu[L]/Leu[L]	360	0.0/0.0227/0.0077	Yes
69329400	1384	rs373052664	N.D.		synonymous	C/T (FWD)	Leu[L]/Leu[L]	361	0.0233/0.0/0.0154	No
69329427	1411	rs139472034	0.0000		synonymous	G/T (FWD)	Gly[G]/Gly[G]	370	0.0/0.0227/0.0077	Yes
69329499	1483	rs370811623	N.D.		synonymous	A/G (FWD)	Val[V]/Val[V]	394	0.0/0.0227/0.0077	Yes
69329514	1498	rs368629288	N.D.		synonymous	C/T (FWD)	Ala[A]/Ala[A]	399	0.0116/0.0/0.0077	Yes
69331208	1546	rs140038894	0.0000		synonymous	C/T (FWD)	Leu[L]/Leu[L]	415	0.0/0.1364/0.0462	Yes
69331328	1666	rs142475179	0.0010		synonymous	A/G (FWD)	Lys[K]/Lys[K]	455	0.0814/0.0/0.0539	Yes
69335070	1735	rs151238781	0.0000		synonymous	A/G (FWD)	Ala[A]/Ala[A]	478	0.0116/0.0/0.0077	No
69335139	1804	rs367755214	N.D.		synonymous	A/G (FWD)	Ser[S]/Ser[S]	501	0.0116/0.0/0.0077	No
69339785	1888	rs373341231	N.D.		synonymous	C/T (FWD)	Thr[T]/Thr[T]	529	0.0116/0.0/0.0077	Yes
69339821	1924	rs149039817	0.0040	0.0018	synonymous	C/T (FWD)	Ile[I]/Ile[I]	541	0.0116/0.4773/0.1693	Yes
69339833	1936	rs141322196	0.0000		synonymous	C/T (FWD)	Ile[I]/Ile[I]	545	0.0233/0.0455/0.0308	Yes
69340248	2059	rs148180732	0.0000		synonymous	C/T (FWD)	His[H]/His[H]	586	0.0116/0.0/0.0077	Yes
69340251	2062	rs368774817	N.D.		synonymous	A/G (FWD)	Lys[K]/Lys[K]	587		Yes
69341328	2092	rs142701949	0.0000		synonymous	A/G (FWD)	Gln[Q]/Gln[Q]	597	0.0233/0.0/0.0154	Yes
69341337	2101	rs373010098	N.D.		synonymous	C/T (FWD)	Phe[F]/Phe[F]	600	0.0233/0.0/0.0154	Yes
69341382	2146	rs143553877	0.0020		synonymous	C/T (FWD)	Ala[A]/Ala[A]	615	0.0116/0.2045/0.0769	Yes
69347675	2164	rs145743798	0.0100	0.0051	synonymous	C/T (FWD)	Gly[G]/Gly[G]	621	0.349/0.0909/0.2616	Yes
69347678	2167	rs370279522	N.D.		synonymous	C/T (FWD)	Arg[R]/Arg[R]	622	0.0/0.0227/0.0077	Yes
69347690	2179	rs139897974	0.0000		synonymous	A/G (FWD)	Leu[L]/Leu[L]	626	0.0/0.0227/0.0077	Yes
69347723	2212	rs375324453	N.D.		synonymous	C/T (FWD)	Asn[N]/Asn[N]	637	0.0116/0.0/0.0077	Yes
69347732	2221	rs149843858	0.0010		synonymous	A/G (FWD)	Lys[K]/Lys[K]	640	0.0/0.0455/0.0154	Yes
69347807	2296	rs139781334	0.0000		synonymous	A/G (FWD)	Thr[T]/Thr[T]	665	0.0116/0.0/0.0077	Yes
69349006	2431	rs373378260	N.D.		synonymous	C/T (FWD)	Phe[F]/Phe[F]	710		Yes

*EA: European Ameriacan.

*AA: African American.

Species Comparation for Conservation includes: *Homo sapiens, Bos taurus, Canis lupus familiaris, Oryctolagus cuniculus, Equus caballus, Macaca mulatta, Gorilla gorilla, Ovis aries, Felis catus and Sus scrofa.*

**Table 4:** Demographics of SNP induced mutations in Nox5.

	rs112069106	rs150003957	rs145609289	rs34406284	rs2277552
	A/T (FWD)	A/C (FWD)	C/T (FWD)	A/G (FWD)	C/T (REV)
	M77K	S236R	T253M	W254Ter*	R530H
ASW	0.0082	0.0082	0.0164	0.0738	0.1148
CEU	0.0000	0.0000	0.0000	0.0000	0.0000
CHB	0.0000	0.0309	0.0000	0.0000	0.2165
CHS	0.0000	0.0350	0.0000	0.0000	0.2100
CLM	0.0000	0.0083	0.0000	0.0000	0.0667
FIN	0.0000	0.0323	0.0000	0.0000	0.0538
GBR	0.0000	0.0112	0.0000	0.0000	0.0000
IBS	0.0000	0.0357	0.0000	0.0000	0.0000
JPT	0.0000	0.0225	0.0000	0.0000	0.2079
LWK	0.0000	0.0104	0.0104	0.0625	0.2083
MXL	0.0000	0.0000	0.0000	0.0000	0.0859
PUR	0.0000	0.0091	0.0000	0.0000	0.0455
TSI	0.0000	0.0102	0.0000	0.0000	0.0000
YRI	0.0000	0.0000	0.0227	0.1080	0.1193

ASW: Americans of African Ancestry in SW USA.

CEU: Utah Residence (CEPH) with Western and Northern European Ancestry.

CHB: Han Chineses in Beijing, China.

CHS: Southern Han Chinese.

CLM: Colombians from Medelin, Colombia.

FIN: Finnish in Finland.

GBR: British in England and Scotland.

IBS: Iberian population in Spain.

JPT: Japanese in Tokyo, Japan.

LWK: Luhya in Webuye, Kenya.

MXL: Mexican Ancestry from Los Angeles USA.

PUR: Puerto Ricans from Puerto Rico.

TSI: Toscani in Italia.

YRI: Yoruba in Ibadan, Nigera.

## Discussion

The functional relevance of Nox5 remains enigmatic. Chief obstacles are the absence of Nox5 in the genomes of rats and mice, the lack of genetic knockouts in other species and the inability to selectively target Nox5 with pharmacological tools. Given these constraints, our knowledge of the functional roles of Nox5 has been limited to *in vitro* or *ex vivo* experiments and correlative studies based on expression levels. In human cells, Nox5 has been ascribed numerous roles including the development and capacitation of sperm, smooth muscle proliferation and migration, endothelial cell proliferation and angiogenesis and cancer cell proliferation and resistance to apoptosis [13], [29]. Nox5 expression is also known to be upregulated in development, cancer, and cardiovascular diseases, but whether it influences the pathogenesis of disease is not year clear. Genetic approaches that correlate small variations in human genomes with disease susceptibility such as GWAS have had limited predictive power in complex polygenic diseases. Therefore in the current study we assessed the functional significance of Nox5 by investigating the how SNPs within coding region of Nox5 influence its enzymatic activity as measured by ROS production.

Nox5 can be functionally divided into 3 domains, an N-terminal EF-hand calcium binding domain, a 6 transmembrane spanning middle region that supports 2 heme molecules, and a C-terminal reductase domain that binds NADPH. The elevation of calcium promotes occupation of the N-terminal EF hands of Nox5 and induces a conformational change that exposes a hydrophobic region. Once released, the hydrophobic core binds to a C-terminal Regulatory EF-hand Binding Domain (deemed REFBD) that is thought to relieve intramolecular inhibition and allow electron flow from the C-terminus, through FAD to the heme moieties to enable superoxide production. Outside of this core mechanism for activation, the phosphorylation of Nox5 (S490, T494, S498) and binding of calmodulin can support increased enzyme activity at lower concentrations of calcium. Additional regulators are the molecular chaperones such as hsp90/hsp70 which bind directly to Nox5 and can influence enzyme stability and the production of superoxide versus hydrogen peroxide [31], [36]. There are 5 reported isoforms of Nox5 (α–ε, V1–V5). The major differences between the isoforms lie within the extreme N-terminus and we have shown that only α and β are capable of ROS production. Given the expression of Nox5β in blood vessels and vascular cells and the lack of profound differences in ROS production versus Nox5α, we have focused our study on the β (v2) isoform which is shortest form capable of ROS production and thus changes in Nox5β should also affect the other active isoforms. However, it should be noted that there are additional SNPs in the other, longer, isoforms of Nox5 that could possibly influence activity.

We generated 15 mutations in Nox5 based on SNPs identified in the coding sequence for Nox5β (**Table 2**). Of these, 7 out of 15 mutants had significantly attenuated activity. The amino acid, M77 is predicted to reside between the 2 calcium-binding EF-hands in the N-terminus of Nox5 (Fig. 7). Substitution to the charged residue, lysine (M77K) resulted in a catastrophic loss of both basal and stimulated activity. Further analysis revealed no differences in the level of Nox5 expression, the degree of phosphorylation or the levels of bound hsp90. Alternatively, isolated enzyme assays suggest that the ability of Nox5 to respond to calcium is severely compromised in the M77K mutant. In transfected COS-7 cells, the intracellular distribution of Nox5 was unaltered by the M77K mutation suggesting more direct effects on enzyme function. To further explore the mechanisms involved, we mutated M77 to valine, based on another reported SNP. The M77V is a more conservative mutation and resulted in a different enzymatic profile for Nox5 including a slight reduction in basal activity, moderately reduced calcium-dependent activity but little change in PMA-stimulated activity. The reasons for this are not yet clear. Binding of calmodulin to some of its target proteins is mediated by methionine residues that lie between 2 pairs of EF-hands [37]. Calcium binding to the EF hands of calmodulin induces a conformation change that exposes the hydrophobic methyl groups of methionine residues and enables it to bind with complementary regions on target proteins. It is therefore tempting to speculate that for Nox5, the hydrophobicity provided by M77 enables the calcium-dependent activation of Nox5 via binding to the C-terminal REFBD. This is supported by the lack of activity of the M77K mutant, where substitution of methionine to a charged, basic amino acid, virtually eliminates activity. A more conservative substitution to the hydrophobic valine in place of a hydrophobic methionine yields a partial phenotype of moderately reduced calcium-dependent activity, slightly reduced basal activity and little change in activity in response to PMA. The reduced calcium-dependency of PMA-stimulated Nox5 activity was observed previously by data showing that the ability of PMA-dependent phosphorylation to activate Nox5 can occur in low but not zero calcium [24]. This is compatible with the concept that the substitution of M77 with valine yields a suboptimal hydrophobic amino acid that retards calcium-dependent activation but is less restrictive in response to PMA.

The most common non-synonymous polymorphism encoding L334F had a frequency of 30% and was without changes in expression or activity of Nox5. This residue is predicted to lie in the 4^th^ transmembrane region of Nox5 and is a highly conserved amino acid in Nox5 across species. The substitution of leucine with phenylalanine can be considered a conservative one given their similar properties including hydrophobicity (GeneDoc) and may not substantially change the structure of Nox5. The SNP responsible for P97A yielded a Nox5 protein with modestly increased activity at rest and following stimulation with both ionomycin and PMA and had a relatively high frequency of 2.3%. P97 is predicted to lie close to the 4^th^ EF hand in the N-terminal region of Nox5 but it is not clear how this change could positively influence activity. The K79I (MAF 0.18%) mutation had a modest increase in activity at rest, no change to ionomycin and a more robust increase with PMA stimulation. K79 is predicted to lie in region between the 2 pairs of EF hands and the loss of a charged residue in this area may potentiate phosphorylation-dependent activity through interaction with the C-terminal REFBD.

The inactive mutants had frequencies ranging from 0.23% to 10.6%. In addition to M77K, the S236R, G542R, V689A mutants had no measurable activity and the T253M, R419Q and R530H had significantly diminished (>85%) activity. S236 (1.5% MAF) is predicted to lie within the 2^nd^ transmembrane region and mutation to a charged arginine residue (S236A) may catastrophically alter enzyme topology and function. T253M has a comparatively low frequency (0.37%) and lies within the 1^st^ loop (A) between transmembrane regions and substitution with a hydrophobic methionine may alter the structure of this loop. R419 lies within the C-terminal region just after the last transmembrane loop and before the predicted FAD binding site. Accordingly, mutation to glutamine may alter electron transfer. In addition to amino acid substitutions there were 3 SNPs (1 validated) that resulted in a stop codon and a truncated variant of Nox5. Lacking 1 or more of the 3 domains necessary for superoxide production, these mutants are predicted to be inactive. The SNP responsible for the W254stop mutant predicts a truncated protein without the middle of C-terminal domains that could not function to produce superoxide. It has a frequency of 1.8% and others have speculated that this could result in the equivalent of a Nox5 knockout in individuals homozygous for this SNP although none so far have been identified [29]. The other missense SNP encoding truncated Nox5 variants (E23stop, Q454stop) are also predicted to be inactive based on the loss of regions critical for enzyme activity, but significance is currently limited by the lack of more rigorous validation and frequency data. The SNP encoding the R530H substitution is considerably more common (10.6%) and also encodes a protein with severely compromised enzyme activity (>95% basal/PMA and >95% ionomycin). This residue resides in the C-terminus and given its predicted location after the FAD and before the NADPH binding sites it should theoretically be better tolerated. Evidence for the disruption of a critical region is derived from the activity of another relatively frequent SNP encoding G542R (1.5%) that lies in close proximity. This mutant was catalytically inactive as determined by superoxide production. Individuals homozygous for either of these SNPs are likely to have little to no Nox5 activity and because protein expression is unaffected, they may also function as a dominant negative as has been shown for other inactive variants of Nox5 [38]. The C-terminal V689A is a more conservative substitution and it was surprising that it had such a dramatic effect on enzyme activity particularly as a more significant nearby substitution, K688E had little effect on activity. It is possible that NADPH binding is altered but this remains speculative.

Based on the 1000 genomes database, we have listed the frequencies of 5 SNPs that alter Nox5 activity significantly among 14 populations including Asia, Europe, Africa and the Americas (**Table 4**) [39]–[41]. Striking differences were observed in the demographics for the SNP encoding W254Ter which was found only in individuals in Africa. The SNP encoding R530H was also present in diverse populations with significant frequencies observed in Asians and Africans, lower in South Americans and absent from Europeans. Whether these differences in the distribution of inactive alleles of Nox5 can help provide insight into the significance of Nox5 to human physiology or pathophysiology remains to be determined. There are other reports linking mutations in Nox5 in Hispanics associating with leptin and fat mass and to lipoprotein-associated phospholipase A2 [42], [43]. While both have strong connections to cardiovascular disease, a causal role for Nox5 remains ambiguous and would benefit from more focused investigation on SNPs with clear changes in activity. Putting this data in perspective, the 1000 genomes project revealed that it is unlikely any individual possesses a perfect set of genes. Rather, on average, each person carries between 250 and 300 genetic changes that result in the loss of gene activity. These changes are buffered by the redundancy of two chromosomes and a second set of intact, working genes.

In addition to the non-synonymous SNP identified within Nox5, it remains possible that SNPs that don’t alter the amino acid sequence of Nox5 may exert other effects such as changes in gene expression. While we found evidence for the high conservation of codons containing synonymous SNP across multiple species, future studies will be needed to rigorously identify whether duons exist with the coding region of Nox5 and also whether SNPs can influence Nox5 expression levels by destruction or creation of new duon sequences. A further limitation of the current study is that SNPs outside of exons which are significantly more numerous, may also contribute to the regulation of Nox5 expression.

In conclusion, our study has revealed a number of exonic SNPs within Nox5 that can influence its activity. We did not reveal any prominent gain of function mutants, but showed that several resulted in severely compromised enzyme activity. These mutations have also revealed new information about the enzymatic function of Nox5. In particular, we found that M77 and the region from R540-G542 are critical for ROS production. Future studies are needed to determine whether there are humans with no or low Nox5 activity and therefore if Nox5 is essential in humans or as with rats and mice, dispensable. Further analysis is also needed to determine whether SNPs coding for mutants of Nox5 with compromised activity associate with increased or decreased disease susceptibility in humans.

## Supporting Information

Figure S1 **Subcellular localization of WT and M77K Nox5.** COS-7 cells were transfected with GFP-Nox5 and RFP-NLS (red fluorescent protein attached to a nuclear localization signal, NLS). Live cells were visualized for GFP and RFP using confocal microscopy.
(TIF)
