# Supplementary figures and images for: Impact of Nox5 Polymorphisms on Basal and Stimulus-Dependent ROS Generation
Source: PLoS One. 2014 Jul 3;9(7):e100102. doi: 10.1371/journal.pone.0100102 (PMC4081039; doi:10.1371/journal.pone.0100102)

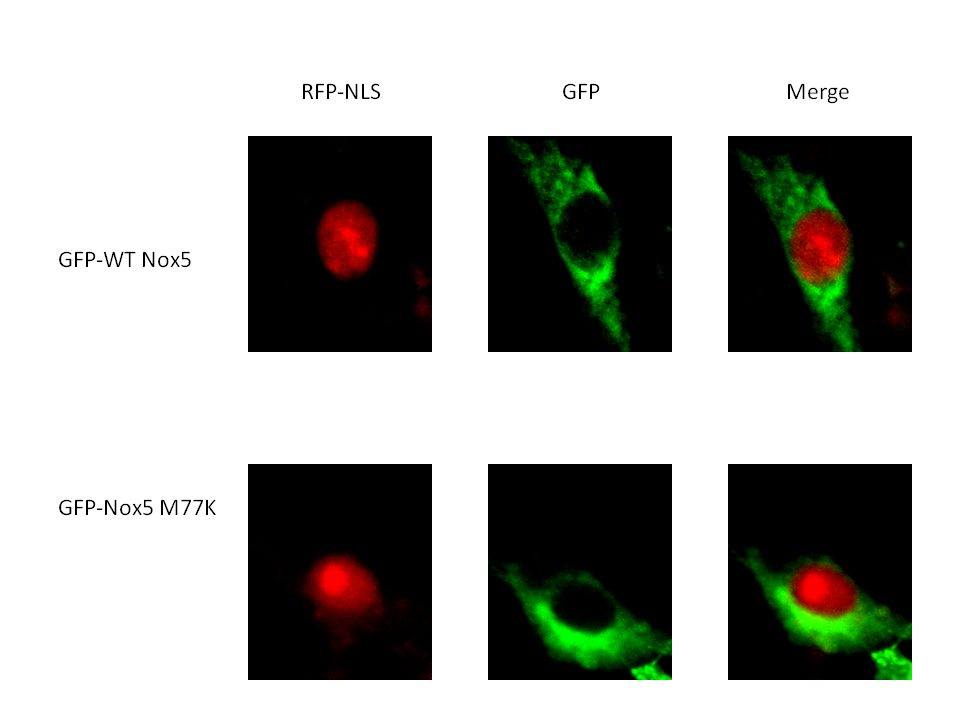

Supplement: Figure S1 — Subcellular localization of WT and M77K Nox5. COS-7 cells were transfected with GFP-Nox5 and RFP-NLS (red fluorescent protein attached to a nuclear localization signal, NLS). Live cells were visualized for GFP and RFP using confocal microscopy. (TIF) [file pone.0100102.s001.tif]
